# Effect of nimodipine combined with atorvastatin calcium on microinflammation and oxidative stress levels in patients with cerebral vasospasm after subarachnoid hemorrhage

**DOI:** 10.12669/pjms.39.2.6721

**Published:** 2023

**Authors:** Ning Gan, Tong-le Jia, Nan Tian, Si-si Liu, Shan Cao

**Affiliations:** 1 Ning Gan, Department of Neurosurgery, Baoding First Central Hospital, Baoding, Hebei, 071000, China; 2 Tong-le Jia, Department of Neurosurgery, Baoding First Central Hospital, Baoding, Hebei, 071000, China; 3 Nan Tian, Department of Neurosurgery, Baoding First Central Hospital, Baoding, Hebei, 071000, China; 4 Si-si Liu. Department of Neurology and Intensive Care, Baoding First Central Hospital, Baoding, Hebei, 071000, China; 5 Shan Cao, Department of Neurology, Baoding First Central Hospital, Baoding, Hebei, 071000, China

**Keywords:** Nimodipine, Atorvastatin calcium, Subarachnoid hemorrhage, Cerebral vasospasm, Oxidative stress

## Abstract

**Objective:**

To evaluate the effect of nimodipine combined with atorvastatin calcium on the micro inflammation and oxidative stress levels in patients with cerebral vasospasm (CVS) after subarachnoid hemorrhage (SAH) and its clinical implications.

**Methods:**

A total of 80 patients with CVS caused by SAH who had been admitted to Baoding First Central Hospital from August 2021 to August 2022 were selected and randomly divided into two groups. The control group underwent conventional symptomatic treatment, while the experimental group was administered nimodipine combined with atorvastatin calcium on the basis of conventional treatment. The changes in the micro inflammatory cytokines and oxidative stress factors in the two groups were compared, as well as the differences in clinical efficacy and incidence of adverse drug reactions.

**Result:**

After treatment, the levels of inflammatory cytokines in the experimental group decreased more significantly than those in the control group (p=0.00). After treatment, the serum levels of oxidative stress factors were obviously higher in the experimental group than in the control group (p=0.00). After treatment, the total efficacy was 77.5% in the experimental group and 55% in the control group, and the difference was statistically significant (p=0.04).

**Conclusions:**

Nimodipine combined with atorvastatin calcium could significantly improve the clinical symptoms in patients with CVS after SAH, which would be beneficial, safe, and effective for the patient’s recovery.

## INTRODUCTION

Subarachnoid hemorrhage (SAH) is a cerebrovascular disease commonly found in clinical practice, with high disability rate and poor prognosis.^1^ The main cause of SAH includes aneurysm, hypertension, arteriosclerosis, and cerebrovascular malformations.^2^ The most common complication of SAH is the secondary cerebral vasospasm (CVS), and data shows that about 30% to 60% of SAH patients are complicated with CVS. Moreover, the high disability rate and fatality rate of SAH are mostly caused by CVS.^3^ Therefore, the treatment of the CVS secondary to SAH is very important in clinical practice. The pathogenesis of CVS after SAH is complicated and remains unclear. There is still no specific drug for its prevention or treatment. At present, although the U.S. Food and Drug Administration (FDA) approved the use of nimodipine only for the treatment of secondary CVS, its efficacy is still not desirable in many patients.^4^ A study has shown that among hydroxymethylglutaryl coenzyme A (HMG-CoA) reductase inhibitors, also known as statins, atorvastatin may exert neuroprotective effects in SAH and can improve CVS by inhibiting the caspase-dependent apoptotic pathway.^5^ In this study, nimodipine combined with atorvastatin calcium was used to treat CVS after SAH. The results suggested that it could significantly improve the clinical symptoms, reduce the inflammatory cytokines, and alleviate the oxidative stress, which would greatly benefit the patients.

## METHODS

A total of 80 patients with CVS caused by SAH who had been admitted to Baoding First Central Hospital from August 2021 to August 2022 were selected and randomly divided into two groups, with 40 cases in each group. Among them, the experimental group consisted of 22 males and 18 females, aged 35 to 69 years old, with an average of 54.74 ± 9.69 years old. The control group consisted of 23 males and 17 females, aged 42 to 67 years old, with an average of 55.21 ± 7.89 years old. General data of the two groups were not significantly different, and the groups were comparable (Table-I). The study was approved by the Institutional Ethics Committee of Baoding First Central Hospital (No.:2022-083; date: 03-08-2022), and written informed consent was obtained from all participants.

**Table-I:** Comparison of general data between the two groups (*χ̅*±*S*) n=40.

Items	Experimental group	Control group	t/χ^2^	p
Age (years old)	54.74±9.69	55.21±7.89	0.24	0.81
Male (n %)	22 (55%)	23 (57.5%)	0.05	0.82
BMI (kg/m^2^)	23.71±3.48	24.20±3.51	0.63	0.53
Causes				
Aneurysm (n %)	16 (40%)	11 (27.5%)	1.40	0.24
Arteriosclerosis (n %)	18 (45%)	20 (50%)	0.20	0.65
Cerebrovascular malformations (n %)	6 (15%)	9 (22.5%)	0.74	0.39
Underlying disease				
Hypertension (n %)	23 (57.5%)	18 (45%)	1.25	0.26
Diabetes (n %)	12 (30%)	14 (35%)	0.23	0.63
Others (n %)	5 (12.5%)	8 (20%)	0.83	0.36

p > 0.05.

### Inclusion criteria:

- Meeting the diagnostic criteria of SAH and CVS;^6^
- Confirmed as SAH by cranial CT within 24 h after admission;
- Having acceptable general state and Hunt-Hess grade ≤ III, and being cooperative;^7^
- Showing signs of neurological positioning, transcranial Doppler ultrasound indicates that the blood flow velocity of the main branches of the skull base > 120 cm/s or that the peak value of the middle cerebral artery blood flow velocity > 200 cm/s, or DSA confirms the presence of CVS;^8^
- The clinical and imaging data are complete;
- The family members of the patients agree to the study protocol and sign the consent form.

### Exclusion criteria:

- Symptomatic CVS;
- Critical conditions, unconsciousness, Hunt-Hess grade > IV, or serious diseases of the heart, brain, and kidney;
- Allergy to the drugs involved in this study;
- Pregnant and nursing women;
- Mental illness, cognitive impairment, or failure to cooperate;
- Recent use of drugs that affect the study results, such as hormones and immunosuppressant’s.

Admitted to the hospital, patients of the control group immediately underwent symptomatic treatment, including reduction of intracranial pressure, dehydration, sedation, anti-infection, and nourishment of brain cells. Patients with aneurysm underwent aneurysm clipping or aneurysm resection. After surgery, the patient’s airway was kept unobstructed, the mental symptoms and blood pressure were controlled, and cerebral edema was prevented or treated. Basic treatments such as blood volume supplementation, maintenance of water and electrolyte, acid-base balance, and nutritional support were conducted. Intravenous infusion of 20ml of nimodipine + 150ml of normal saline was administered once a day, for a total of 14 days. In addition to the treatment of the control group, the experimental group was administered 20 mg of atorvastatin calcium once a day.

### Observation Indicators:

### Micro inflammation indicators:

five ml of peripheral venous blood was drawn from all patients before and 14 days after treatment. Enzyme-linked immunosorbent assay (ELISA) was used to measure the levels of inflammatory cytokines such as tumor necrosis factor (TNF-a), C-reactive protein (CRP), and interleukin-six (IL-6).

### Oxidative stress indicators:

five ml of peripheral venous blood was drawn from all patients before and 14 days after treatment. The supernatant was taken out, and ELISA was used to measure the levels of serum oxidative stress indicators, including superoxide dismutase (SOD) and glutathione Peptide peroxidase (GSH-Px), total antioxidant capacity (TAC), catalase (CAT), and glutathione reductase (GR).

### Efficacy evaluation:

Significantly effective: After treatment, most of the clinical symptoms of the patient disappeared, and the neurological deficit score (NDS) decreased by 46% to 90%; Effective: After treatment, the patient’s clinical symptoms were basically improved, and the NDS decreased by 16% to 45%; Ineffective: After treatment, the patient’s clinical symptoms were not improved, and the NDS decreased by less than 15%. The total effective rate (%) = significantly effective rate + Effective rate.^9^

### Adverse reactions:

The incidence of adverse reactions within 14 days after administration was compared between the two groups.

### Statistical Analysis:

SPSS 20.0 software was used for statistical analysis of all data. The measurement data were expressed as (±S). A two-sample independent t-test was used for comparison of data between groups, while repeated measures analysis of variance was used for analysis of data within the group. χ^2^ test was used for the comparison of rates. P<0.05 was considered statistically significant.

## RESULTS

There was no significant difference in the levels of inflammatory cytokines including TNF-a, CRP, and IL-6 between the two groups before treatment (p> 0.05). After treatment, these indicators were lower than before treatment, and the differences were statistically significant (p=0.00). The above indicators were more significantly reduced in the experimental group than in the control group, and the differences were statistically significant (p=0.00) (Table-II).

**Table-II:** Comparison of inflammatory cytokines between the two groups (*χ̅*±*S*) n=40.

Items		Before treatment*	14 days after treatment, D	t	p
TNF-ɑ (ng/L)	Experimental group D	46.38±6.53	21.30±5.32	18.54	0.00
	Control group D	46.71±7.01	25.36±5.41	13.68	0.00
	t	0.27	7.44		
	p	0.79	0.00		
CRP (mg/L)	Experimental group D	65.38±7.26	11.53±3.27	42.77	0.00
	Control group D	63.89±6.73	20.61±4.06	34.83	0.00
	t	0.95	11.02		
	p	0.34	0.00		
IL-6 (ng/L)	Experimental group Δ	17.69±5.47	8.72±2.63	9.35	0.00
	Control group D	18.05±6.03	13.46±3.85	4.06	0.00
	t	0.28	6.43		
	p	0.78	0.00		

* p< 0.05

After treatment, the comparison of the antioxidants between the two groups suggested that the serum levels of SOD, TAC, and CAT were significantly higher in the experimental group than in the control group, and the differences were statistically significant (p=0.00). There was no significant difference in the levels of GSH-Px and GR between the two groups (p>0.05) (Table-III).

**Table-III:** Comparison of antioxidants between the two groups (*χ̅*±*S*±*S*) n=40

Group	SOD (U/ml) *	GSH-Px (U/ml)	TAC (U/ml) *	CAT (U/ml) *	GR (U/ml)
Experimental	85.31±7.32	340.13±31.27	17.88±2.25	13.76±2.02	128.45±17.27
Control	62.18±7.13	337.39±29.76	12.02±1.77	8.14±1.06	126.53±11.32
t	17.53	0.49	15.86	19.08	0.72
p	0.00	0.62	0.00	0.00	0.47

* p< 0.05

The comparison of efficacy between the two groups is shown in Table-IV. The results showed that after treatment, the total effective rate was 77.5% in the experimental group and 55% in the control group. The effective rate of treatment was higher in the experimental group than in the control group, and the difference was statistically significant (p=0.04).

**Table-IV:** Comparison of efficacy between the two groups (*χ̅*±*S*) n=40.

Group	Significantly effective	Effective	Ineffective	Total effective rate*
Experimental	17	14	9	31 (77.5%)
Control	13	9	18	22 (55%)
c^2^				4.17
p				0.04

* p< 0.05.

The prevalence of adverse drug reactions within 14 days after treatment was 35% in the experimental group and 22.5% in the control group, and the difference was not statistically significant (p=0.21) (Table-V).

**Table-V:** Comparison of adverse drug reactions after treatment between the two groups (*χ̅*±*S*) n=40.

Group	Dizziness	Gastrointestinal reaction	Headache	PLT reduction	Flushing	Hypotension	Total incidence*
Experimental	2	1	2	3	2	4	14 (35%)
Control	1	3	0	2	0	3	9 (22.5%)
c^2^							1.53
p							0.21

p> 0.05.

## DISCUSSION

According to statistics the incidence of CVS secondary to SAH is about 32% to 66%.^10^ The diagnosis is mainly based on imaging and neurological examinations. The clinical manifestations of the patient include consciousness disorders and neurological dysfunction such as dyskinesia, sensory disturbances, and aphasia.^11^ A large-scale meta-analysis showed that the peak incidence of CVS occurs in one to three weeks after SAH.^12^ It is a major cause of disability and death of SAH patients. Active and effective diagnosis and treatment are key factors for the prognosis of the patients. Nimodipine is a dihydropyridine calcium channel antagonist, which can directly enter the central nervous system through the blood-brain barrier (BBB) to improve the state of vasospasm and increase the blood flow of brain tissues.^13^ But it also affects other smooth muscle cells throughout the body, causing certain side effects.^14^

Inflammatory responses are the body’s normal reaction to harmful substances from the outside world and help repair damaged tissues and organs. Studies have shown that the white blood cells and inflammatory cytokines in the blood of SAH patients are significantly elevated, and the inflammatory response as a mechanism is involved in the pathogenic process of CVS after SAH.^15^ Animal experiments have shown that the occurrence of CVS after SAH and the degree of harm to patients are significantly related to the concentrations of inflammatory cytokines such as TNF-a and IL-6.^16^

Moreover, the higher the concentrations of inflammatory cytokines in the cerebral ventricles after hemorrhage, the higher the occurrence of CVS and the worse the prognosis. The increased level of superoxide anion in cerebrospinal fluid is closely related to the occurrence of CVS. Karaoglan et al. reported that the therapeutic measures to inhibit or eliminate free radicals in SAH animal models can alleviate CVS, with an effective rate of 70%.^17^ It has been confirmed that inhibiting the oxidative stress response can prevent cell apoptosis and the destruction of the BBB.^18^

As a statin, atorvastatin can improve the bioavailability of endogenous nitric oxide and up-regulate endothelial nitric oxide synthase, which is used to prevent aneurysm-type CVS after SAH. At the same time, cholesterol-lowering drugs and antioxidant properties with multiple effects can inhibit the oxidative stress response by reducing the production of ROS. Xu et al. and colleges believe that have a wide range of immunomodulatory and anti-inflammatory properties, and can effectively regulate nerve injury after changing the mechanism of peripheral leukocyte invasion and microglia/macrophage alternate polarization.^19^ Animal experiments confirmed that the application of atorvastatin after treatment could significantly reduce the apoptosis of rabbit hippocampal neurons in SAH.^20^ In addition to promoting anti-apoptotic signals, atorvastatin treatment can also reduce CVS and mediate the structural and functional remodeling of vascular endothelial cells. Chen et al. believed that the combined use of atorvastatin and nimodipine could reduce the incidence of CVS and cerebral infarction after SAH.^21^ It can considerably benefit the SAH patients.

Our study confirmed that nimodipine combined with atorvastatin calcium could reduce the levels of inflammatory cytokines including TNF-a, CRP, and IL-6 in patients with CVS after SAH more significantly than the conventional treatment in the control group (p=0.00). After treatment, the serum levels of SOD, TAC, and CAT were significantly higher in the experimental group than in the control group, and the difference was statistically significant (p=0.00). After treatment, the total efficacy was 77.5% in the experimental group and 55% in the control group, and the difference was statistically significant (p=0.04). The incidence of adverse drug reactions within 14 days after treatment was 35% in the experimental group and 22.5% in the control group, and the difference was not statistically significant (p=0.21).

### Limitations:

It includes the small sample size and short follow-up time. As a result of the small sample size, SAH patients caused by different reasons were not investigated separately. Moreover, nimodipine was administered only by intravenous infusion, and the oral route of administration was not included in the study for comparison. In future clinical work, we will further expand the sample size, and SAH cases induced by different causes such as aneurysm, hypertension, arteriosclerosis, and cerebrovascular malformations should be compared separately. In addition, the differences in the results under different administration routes should be explored, in order to comprehensively evaluate the advantages and disadvantages of the treatment protocol.

## CONCLUSIONS

Nimodipine combined with atorvastatin calcium could significantly improve the clinical symptoms reduce the inflammatory cytokines, and alleviate the oxidative stress in patients with CVS after SAH, while there was no significant increase in the adverse drug reactions, which would be beneficial, safe, and effective for the patient’s recovery.

### Authors’ contributions:

**NG and TJ:** Carried out the studies, participated in collecting data, and drafted the manuscript.

**NT and** **SL:** Performed the statistical analysis and participated in its design.

**SC:** Participated in acquisition, analysis, or interpretation of data and draft the manuscript.

All authors read and approved the final manuscript.
